# Knowledge, Attitudes, and Practices About Sarcopenia in Adults With Type 2 Diabetes: A Cross‐Sectional Survey

**DOI:** 10.1155/jdr/3057863

**Published:** 2026-04-23

**Authors:** Xiaodi Sun, Wen Liu, Song Han, Min Wang, Zengqiang Liu, Weidong Jiang, Aiqiong Qin

**Affiliations:** ^1^ Department of Geriatric Medicine, The Second Qilu Hospital of Shandong University, Jinan, 250033, China; ^2^ Department No. 29 Comprehensive Medical, Emergency General Hospital, Beijing, 100028, China

**Keywords:** cross-sectional study, diabetes mellitus, knowledge attitude practice, sarcopenia

## Abstract

**Background:**

Sarcopenia is a frequent and clinically significant complication in individuals with type 2 diabetes mellitus (T2DM) and is linked to unfavorable outcomes, including loss of physical function and disability. Yet, little is known about how patients themselves understand and manage this condition, and data on their knowledge, attitudes, and practices (KAP) toward sarcopenia are particularly scarce among people with T2DM in China. Therefore, this study sought to investigate T2DM patients’ KAP related to sarcopenia.

**Methods:**

This cross‐sectional survey enrolled adults with T2DM, recruited consecutively from both outpatient clinics and inpatient wards of a tertiary hospital in China (November 2022–April 2023). Participants completed a self‐administered KAP questionnaire on sarcopenia (12 knowledge items scored 0–12; 12 attitude items scored 12–60; 7 scored practice items scored 7–35, plus 3 open‐ended practice questions). Descriptive statistics were used to summarize KAP scores, Spearman correlation assessed associations among KAP domains, and structural equation modeling was employed to test the hypothesized pathways.

**Results:**

A total of 475 completed questionnaires were included in the analysis. Mean scores for KAP were 4.55 ± 1.98 (range: 0–12), 47.27 ± 2.89 (range: 12–60), and 19.63 ± 3.51 (range: 7–35), respectively. Spearman correlation analysis indicated weak but statistically significant positive associations between each pair of domains: knowledge and attitude (*r* = 0.222), knowledge and practice (*r* = 0.375), and attitude and practice (*r* = 0.485) (all *p*  < 0.001). In the structural equation model, knowledge was positively associated with both attitude and practice, and attitude was likewise associated with practice (β = 0.160, *p* = 0.012; β = 0.281, *p* = 0.003; β = 0.267, *p* = 0.006). Despite the high‐risk nature of this population, only 2.53% of participants reported having ever received a clinical diagnosis of sarcopenia, indicating that sarcopenia was rarely documented in routine diabetes care in this cohort.

**Conclusions:**

Patients with T2DM demonstrated limited knowledge but overall favorable attitudes and moderate practice behaviors related to sarcopenia. The low proportion of sarcopenia identified by clinicians highlights limited detection in routine care and supports more structured education and opportunistic screening during diabetes visits.

## 1. Introduction

Sarcopenia is defined by a progressive reduction in skeletal muscle mass, strength, and function and has become a prevalent condition with substantial economic implications. It is linked to a range of adverse outcomes—including falls, functional disability, hospitalization, and higher mortality—that collectively exert a marked negative impact on patients’ quality of life [1–4]. Individuals with type 2 diabetes mellitus (T2DM) are at heightened risk of developing sarcopenia as a consequence of diabetes‐related metabolic disturbances, insufficient nutritional intake, and insulin resistance, all of which contribute to muscle wasting [5]. The prevalence of sarcopenia is substantially higher among individuals with T2DM than in those without diabetes. In the elderly population, the prevalence rates range from 15% to 44% [2, 6]. For instance, a particular study indicated that 14.8% of T2DM patients aged 60 years and older exhibited sarcopenia, while the prevalence among healthy controls was significantly lower at 11.2% [7]. As the global population ages and dietary patterns evolve, the prevalence of T2DM is escalating. This phenomenon is contributing to a growing cohort of patients experiencing both sarcopenia and an increased risk of disability and mortality [8]. In contrast to other prevalent complications associated with diabetes, including retinopathy, nephropathy, and cardiovascular disease, sarcopenia is often neglected or underestimated in clinical practice, which may lead to insufficient routine screening and a potential widespread underdiagnosis of sarcopenia among patients with T2DM, as many cases remain unidentified until functional decline becomes evident [6, 7].

Recent longitudinal studies have elucidated a bidirectional relationship between sarcopenia and T2DM. A 7‐year cohort study conducted in China demonstrated that the presence of possible sarcopenia increases the risk of developing new‐onset T2DM [9]. This association underscores the imperative for early intervention, particularly through lifestyle modifications, to prevent or delay the onset of both conditions. This necessity is particularly pronounced among older patients, who bear a disproportionately greater burden, as well as middle‐aged patients, who may derive substantial benefits from preventive measures. By 2050, more than 30% of China’s population is projected to be aged ≥60 years, and China accounts for approximately one‐quarter of the global diabetes burden [10]. In Shandong Province, an estimated 9.2 million adults were living with diabetes in 2018, indicating a substantial local disease burden in the study setting [11]. Addressing sarcopenia within this demographic constitutes not only a national priority for healthy aging but also an issue with global health implications, as China’s strategies and evidence may inform international approaches to managing sarcopenia in high‐risk diabetic populations [12].

The effective management of sarcopenia necessitates a dual approach encompassing both medical interventions and active patient engagement. This engagement largely depends on patients’ knowledge, attitudes, and practices (KAP) related to the condition. According to the KAP framework, knowledge provides the basis for behavior change, whereas attitudes and beliefs drive and sustain that change [13, 14]. Studies have demonstrated that evaluating KAP among T2DM patients can illuminate areas for enhancement in disease management [3]. For instance, a study of Korean patients revealed a sarcopenia prevalence of 15.7% among T2DM patients, compared to 6.9% in non‐diabetic controls, further accentuating the burden of this condition within this population [1].

Despite the acknowledged burden of sarcopenia and the critical nature of patient engagement, comprehensive research on the KAP of T2DM patients regarding sarcopenia is limited, particularly in China. While some studies have explored the general public’s understanding [15] or specific aspects such as exercise perceptions among T2DM patients with sarcopenia [16], a notable gap persists in understanding the broader KAP related to sarcopenia in this population. Research conducted by Che et al. [16] highlighted fragmented and unreliable knowledge regarding exercise, particularly among older, less educated, and lower‐income individuals. However, this research primarily focused on exercise perceptions rather than a comprehensive evaluation of KAP concerning sarcopenia, leaving critical inquiries about diagnostic awareness and the translation of positive attitudes into actionable behaviors unanswered. In light of the urgent need for T2DM patients to engage in sarcopenia management and the current lack of comprehensive understanding of their awareness and beliefs regarding the condition. Therefore, this study sought to evaluate KAP related to sarcopenia among adults with T2DM in China through a cross‐sectional questionnaire survey. We further examined the associations among KAP domains and tested a hypothesized pathway model using structural equation modeling.

## 2. Materials and Methods

### 2.1. Study Design and Participants

This cross‐sectional investigation was carried out between November 2022 and April 2023 among patients with T2DM receiving care at the Second Hospital of Shandong University [17]. The study protocol was approved by the Second Medical Ethics Committee of Shandong University, and written informed consent was obtained from all participants prior to questionnaire administration.

### 2.2. Inclusion and Exclusion Criteria

Adults aged 18 years or older with a confirmed diagnosis of T2DM and sufficient ability to complete the questionnaire independently were eligible for inclusion. Patients with severe cognitive impairment or communication difficulties that precluded completion of the questionnaire were excluded. This study excluded invalid submissions flagged by platform‐based checks (e.g., duplicated responses).

### 2.3. Procedures

The questionnaire was developed with reference to AWGS 2019 [18], EWGSOP2 2019 [3], the 2021 Chinese Expert Consensus on the Diagnosis and Treatment of Sarcopenia in the Elderly [4], the 2021 Chinese Expert Consensus on Core Messages for the Prevention of Sarcopenia in Older Adults [12], and those questionnaire designs reported by other researchers [19, 20], and then revised with input from three senior experts. After the completion of the questionnaire design, a total of 76 questionnaires were collected for the pilot study, and reliability testing yielded a Cronbach’s *α* of 0.820, indicating good internal consistency. The pilot sample size was chosen pragmatically to allow preliminary assessment of item clarity and internal consistency before the formal survey. Pilot participants were recruited independently and were excluded from the final analysis dataset.

The final questionnaire comprised four sections: demographic characteristics and three KAP dimensions (knowledge, attitudes, and practices). The knowledge section included 12 items, each scored 1 for a correct response and 0 for an incorrect or “uncertain” response, yielding a total score range of 0–12. The attitudes section contained 12 statements rated on a five‐point Likert scale from “strongly agree” (5) to “strongly disagree” (1), with total scores ranging from 12 to 60. Of these items, A1–A8 and A10 reflected positive attitudes and were scored from 5 (“strongly agree”) to 1 (“strongly disagree”), whereas A9 and A11–A12 were negatively worded and reverse‐scored from 1 to 5. The practices section included 10 items; seven were closed‐ended items rated on a five‐point Likert scale from “always” (5) to “never” (1), producing a quantitative practice score between 7 and 35, and the remaining three were open‐ended questions that were summarized descriptively and not incorporated into the practice score. These open‐ended responses were summarized descriptively to capture qualitative insights on treatment intention, perceived barriers/concerns, and willingness to receive education.

Questionnaires were self‐administered by outpatients and inpatients with T2DM, using either paper forms or an electronic version hosted on the Wenjuanxing platform (Wenjuanxing Tech Co., Ltd., Changsha, China) accessed via a QR code. For the electronic survey, participants were required to select “I agree to participate in this study” before proceeding to the items. All responses were collected anonymously. To prevent duplicate submissions, an IP restriction was implemented so that each IP address could complete the questionnaire only once.

### 2.4. Sample Size

The required sample size was estimated using the standard formula for cross‐sectional surveys [21]: $N={\left(\frac{Z_{12-\alpha /}}{\delta }\right)}^{2}\times p\times \left(1-p\right)$. Assuming a 95% confidence level 95% (*Z*_1−*α*/2_ = 1.96), a margin of error (δ) of 5%, and a conservative expected prevalence (p) of 50% to yield the largest sample size, the minimum number of participants needed was 385. Allowing for an anticipated nonresponse rate of 15%, the final target sample size was increased to ~454 individuals.

### 2.5. Statistical Analysis

Stata 17.0 (Stata Corporation, College Station, TX, USA) was used for all statistical analyses. Descriptive analyses were performed to examine the demographic characteristics of the respondents, alongside their KAP scores, with means and standard deviations reported to encapsulate the data. A score exceeding 70% of the maximum possible value was classified as good, scores between 50% and 70% were classified as moderate, and scores below 50% were classified as poor [22]. Response frequencies for each item were expressed as n (%), stratified by various demographic characteristics. For continuous variables with an approximately normal distribution, independent‐samples *t* tests or one‐way analysis of variance (ANOVA) were used for group comparisons. For skewed variables, the Wilcoxon–Mann–Whitney or Kruskal–Wallis tests were applied. Responses to the three open‐ended practice items were analyzed separately and summarized descriptively, and were not included in the calculated practice score. Spearman correlation analysis was performed to explore associations among KAP scores.

A structural equation model (SEM) was specified and estimated in IBM SPSS Amos 27.0 to examine the hypothesized relationships: that knowledge influences attitudes; that knowledge affects practices both directly and indirectly via attitudes; that attitudes have a direct effect on practices; that regular exercise is related to both attitudes and practices; that sarcopenia status is associated with knowledge and practices; and that monthly income is related to practices. In addition, potential effects of sociodemographic characteristics on KAP were incorporated into the model.

Confirmatory factor analysis (CFA) was first conducted to evaluate the construct validity of the KAP questionnaire before testing the structural paths. Model evaluation considered standardized factor loadings, critical ratios (C.R.), and several goodness‐of‐fit indices, including the chi‐square to degrees of freedom ratio (CMIN/DF, with values of 1–3 indicating excellent fit and 3–5 acceptable fit), Normed Fit Index (NFI > 0.80), Incremental Fit Index (IFI > 0.80), Tucker–Lewis Index (TLI > 0.80), and Comparative Fit Index (CFI > 0.80). A two‐sided *p* value < 0.05 was regarded as statistically significant.

## 3. Results

### 3.1. Demographic Characteristics and KAP Scores

A total of 577 questionnaires were obtained for this study, of which 475 were valid after removing 102 submissions during data cleaning, with an effectiveness rate of 82.32%. Among them, there were 261 men, accounting for 54.95%. More than half of the participants were aged over 60 years, accounting for 56.42%. 73.84% of participants had a physical exercise routine, while 89.47% of participants reported a weekly cumulative duration of moderate‐to‐vigorous physical activity of less than 60 min, which is generally below commonly recommended activity levels. Approximately 80% of participants did not use walking aids, did not experience a fall in the last 1 year, and did not have balance difficulties. The mean scores for KAP were 4.55 ± 1.98 (range: 0–12), 47.27 ± 2.89 (range: 12–60), and 19.63 ± 3.51 (range: 7–35), respectively (Table 1).

**Table 1:** Demographic characteristics.

Variables	*N* (%)	Knowledge scores		Attitude scores		Practice scores	
		Mean ± SD	*p*	Mean ± SD	*p*	Mean ± SD	*p*
Total	475	4.55 ± 1.98	—	47.27 ± 2.89	—	19.63 ± 3.51	—
Gender	—	—	0.024	—	0.036	—	0.144
Male	261 (54.95)	4.74 ± 1.94	—	47.52 ± 2.68	—	19.85 ± 3.31	—
Female	214 (45.05)	4.32 ± 2.02	—	46.96 ± 3.11	—	19.37 ± 3.73	—
Age	—	—	0.458	—	0.241	—	0.410
44 years old and below	52 (10.95)	4.23 ± 1.81	—	47.87 ± 1.95	—	19.77 ± 2.99	—
45–59 years old	155 (32.63)	4.62 ± 2.04	—	47.08 ± 3.05	—	19.91 ± 3.17	—
60 years old and above	268 (56.42)	4.57 ± 1.99	—	47.26 ± 2.95	—	19.45 ± 3.78	—
Marital status	—	—	<0.001	—	0.001	—	<0.001
Married	427 (90.27)	4.65 ± 1.89	—	47.41 ± 2.81	—	19.95 ± 3.10	—
Other (including unmarried, divorced, widowed)	46 (9.73)	3.57 ± 2.54	—	45.98 ± 3.30	—	16.57 ± 5.25	—
Education	—	—	0.021	—	0.058	—	<0.001
Middle school and below	231 (48.63)	4.30 ± 2.05	—	46.98 ± 3.05	—	19.04 ± 3.99	—
High school and Technical secondary school	134 (28.21)	4.87 ± 1.98	—	47.37 ± 2.64	—	19.90 ± 3.03	—
Undergraduate and above	110 (23.16)	4.68 ± 1.79	—	47.76 ± 2.79	—	20.55 ± 2.65	—
Monthly income	—	—	<0.001	—	<0.001	—	<0.001
<5000 yuan	96 (20.21)	3.69 ± 2.47	—	44.16 ± 3.39	—	16.66 ± 4.56	—
5000–10,000 yuan	312 (65.68)	4.76 ± 1.61	—	48.09 ± 2.01	—	20.39 ± 2.56	—
>10000 yuan	67 (14.11)	4.79 ± 2.43	—	47.93 ± 2.62	—	20.37 ± 3.38	—
Retirement	—	—	0.415	—	0.026	—	0.043
Yes	288 (60.63)	4.49 ± 2.00	—	47.03 ± 3.03	—	19.37 ± 3.74	—
No	187 (39.37)	4.64 ± 1.97	—	47.64 ± 2.64	—	20.04 ± 3.08	—
Frequent smoker	—	—	0.700	—	0.094	—	0.248
Yes	44 (9.26)	4.77 ± 2.68	—	46.61 ± 2.76	—	18.91 ± 3.73	—
No	382 (80.42)	4.52 ± 1.90	—	47.41 ± 2.85	—	19.76 ± 3.36	—
Quit smoking	49 (10.32)	4.61 ± 1.93	—	46.76 ± 3.24	—	19.31 ± 4.33	—
Frequent alcohol drinker	—	—	0.534	—	0.647	—	0.106
Yes	30 (6.32)	4.93 ± 3.12	—	47.07 ± 2.99	—	18.33 ± 4.41	—
No	394 (82.95)	4.52 ± 1.94	—	47.32 ± 2.88	—	19.74 ± 3.43	—
Quit drinking	51 (10.74)	4.59 ± 1.47	—	46.96 ± 2.98	—	19.59 ± 3.42	—
Physical exercise routine	—	—	<0.001	—	<0.001	—	<0.001
Yes	350 (73.84)	4.77 ± 1.70	—	47.91 ± 2.32	—	20.71 ± 2.33	—
No	124 (26.16)	3.93 ± 2.53	—	45.44 ± 3.51	—	16.60 ± 4.42	—
Weekly average time of moderate to high intensity exercise	—	—	<0.001	—	<0.001	—	0.756
Less than 60 min	425 (89.47)	4.41 ± 1.79	—	47.51 ± 3.12	—	19.62 ± 3.39	—
More than 60 min	50 (10.53)	5.74 ± 2.95	—	45.20 ± 3.12	—	19.78 ± 4.46	—
Walking aids (e.g., crutches, wheelchair, etc.)	—	—	<0.001	—	<0.001	—	<0.001
Yes	81 (17.05)	3.75 ± 2.11	—	45.79 ± 3.35	—	17.02 ± 4.83	—
No	394 (82.95)	4.71 ± 1.92	—	47.57 ± 2.69	—	20.17 ± 2.90	—
Experienced a fall in the last 1 year	—	—	<0.001	—	<0.001	—	<0.001
Yes	78 (16.42)	3.68 ± 2.28	—	45.31 ± 3.43	—	17.06 ± 4.42	—
No	397 (83.58)	4.72 ± 1.88	—	47.65 ± 2.61	—	20.14 ± 3.06	—
Balance difficulties	—	—	<0.001	—	<0.001	—	<0.001
Yes	98 (20.63)	3.77 ± 2.26	—	45.45 ± 3.54	—	17.17 ± 4.50	—
No	377 (79.37)	4.75 ± 1.86	—	47.74 ± 2.50	—	20.27 ± 2.88	—
Diabetes complications (e.g., diabetic foot, diabetic nephropathy, diabetic retinopathy, etc.)	—	—	<0.001	—	0.009	—	0.058
Yes	215 (45.26)	4.15 ± 1.72	—	46.89 ± 3.32	—	19.30 ± 3.69	—
No	260 (54.74)	4.88 ± 2.13	—	47.58 ± 2.44	—	19.91 ± 3.33	—
BMI (body mass index)	—	—	<0.001	—	<0.001	—	<0.001
<18.5 (light)	13 (2.75)	2.92 ± 2.06	—	43.77 ± 4.51	—	14.23 ± 4.44	—
18.5–23.9 (normal)	297 (62.92)	4.52 ± 1.77	—	47.76 ± 2.60	—	20.19 ± 2.86	—
24–27.9 (overweight)	140 (29.66)	4.86 ± 2.23	—	47.01 ± 2.84	—	19.21 ± 4.08	—
>28 (obese)	22 (4.66)	3.64 ± 2.24	—	44.50 ± 2.81	—	18.50 ± 3.91	—
Diagnosed with sarcopenia by the physician	—	—	0.616	—	0.597	—	0.200
Yes	12 (2.53)	4.83 ± 1.99	—	46.83 ± 3.41	—	20.92 ± 5.88	—
No	463 (97.47)	4.54 ± 1.99	—	47.28 ± 2.88	—	19.60 ± 3.43	—

The Kaiser–Meyer–Olkin (KMO) measure was 0.902, and Bartlett’s test of sphericity was significant (*p*  < 0.001). CFA indicated an acceptable model fit, with IFI = 0.880, CMIN/DF = 3.571, TLI = 0.861, and CFI = 0.880.

### 3.2. Distribution of Knowledge, Attitude and Practice

The study showed that most respondents with T2DM had at least a basic understanding of what sarcopenia is and how it relates to both aging and diabetes. Specifically, 86.11% accurately acknowledged that diabetic patients are at an elevated risk for developing sarcopenia, while 83.79% identified the elderly as a particularly vulnerable demographic. This reflects a commendable comprehension of fundamental concepts. However, knowledge regarding diagnostic methods was insufficient, as only 16.63% recognized that gait assessment is essential for screening sarcopenia, underscoring the necessity for enhanced educational initiatives on diagnostic techniques. Furthermore, awareness of the heightened risk of cardiovascular disease associated with sarcopenia was limited, with only 40.21% providing correct responses (Table S1).

The majority of respondents demonstrated either a neutral or positive attitude toward the prevention and management of sarcopenia. For instance, 98.25% (A1) believed that maintaining muscle health is vital for overall well‐being, and 87.53% (A3) acknowledged the significance of appropriate physical activity for muscle health. Nonetheless, 73.25% (A9) expressed apprehensions regarding potential treatment side effects, and 12.06% (A6) disagreed with the notion that they belonged to a high‐prevalence group, suggesting some degree of denial or lack of awareness (Table S2).

Moreover, a significant proportion of respondents reported rarely or never engaging in sarcopenia‐related behaviors (P1 : 94.32%, P2 : 96.85%, P3 : 96.21%), which correspond to three practice items assessing the frequency of sarcopenia‐related behaviors as defined in our questionnaire (item wording shown in Supporting Information Table 3); nevertheless, they demonstrated a high level of adherence to medical recommendations, with 88.84% (P6) consistently following their physician’s advice for managing type 2 diabetes and sarcopenia. In terms of treatment preferences (P8), 74.11% favored a combination of moderate‐to‐high‐intensity exercise and a high‐protein diet, indicating a preference for a comprehensive approach. Concerns regarding treatment (P9) were significant, with 68.21% citing cost, 65.89% indicating distance, and 44.00% referencing lengthy treatment durations as substantial barriers to care (Table S3).

### 3.3. Spearman Correlation Analysis

Spearman correlation coefficients indicated weak to moderate positive relationships between knowledge and attitude (*r* = 0.222, *p*  < 0.001), knowledge and practice (*r* = 0.375, *p*  < 0.001), and attitude and practice (*r* = 0.485, *p*  < 0.001) (Table 2).

**Table 2:** Spearman correlation analysis.

Dimension	Knowledge	Attitudes	Practice
Knowledge	1	—	—
Attitude	0.222 (*p* < 0.001)	1	—
Practice	0.375 (*p* < 0.001)	0.485 (*p* < 0.001)	1

### 3.4. Structural Equation Model Analysis

**Figure 1:**
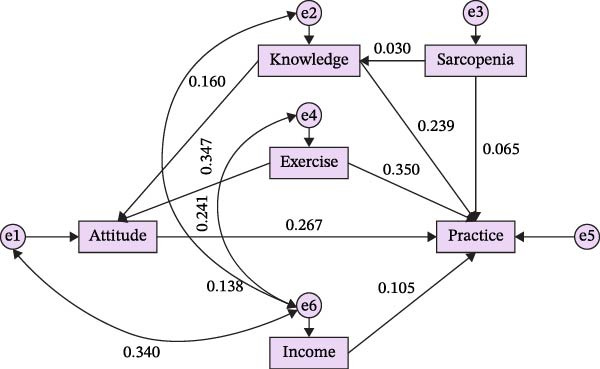
Structure equation model on KAP of adults with type 2 diabetes regarding sarcopenia based on the theory of planned behavior. Standardized path coefficients were presented.

The SEM showed an acceptable overall fit, with CMIN/DF = 4.618, NFI = 0.961, IFI = 0.969, TLI = 0.882, and CFI = 0.969 (Table S4). In this model, knowledge had significant positive effects on both attitude (β = 0.160, *p* = 0.012) and practice (β = 0.281, *p* = 0.003), and attitude exerted a significant direct effect on practice (β = 0.267, *p* = 0.006) (Figure 1, Table 3).

**Table 3:** Structural equation modeling analysis.

Model paths	Standardized total effect		Standardized drect effect		Standardized indirect effect	
	β (95% CI)	*p*	β (95% CI)	*p*	β (95% CI)	*p*
Sarcopenia → knowledge	0.030 (−0.049,0.128)	0.372	0.030 (−0.049,0.128)	0.372	—	—
Sarcopenia → atitude	0.005 (−0.006,0.031)	0.285	—	—	0.005 (−0.006,0.031)	0.285
Exercise → attitude	0.347 (0.249,0.445)	0.012	0.347 (0.249,0.445)	0.012	—	—
Knowledge → attitude	0.160 (0.038,0.251)	0.012	0.160 (0.038,0.251)	0.012	—	—
Sarcopenia → practice	0.074 (−0.079,0.184)	0.313	0.065 (−0.085,0.182)	0.392	0.008 (−0.011,0.037)	0.296
Exercise → practice	0.443 (0.320,0.542)	0.014	0.350 (0.206,0.431)	0.032	0.093 (0.056,0.149)	0.006
Knowledge → practice	0.281 (0.180,0.416)	0.003	0.239 (0.131,0.378)	0.003	0.043 (0.009,0.080)	0.009
Income → practice	0.105 (0.019,0.224)	0.013	0.105 (0.019,0.224)	0.013	—	—
Attitude → practice	0.267 (0.168,0.371)	0.006	0.267 (0.168,0.371)	0.006	—	—

## 4. Discussion

This cross‐sectional study revealed that patients with T2DM possess limited knowledge regarding sarcopenia, even though their attitudes were generally positive and their practice behaviors were at a moderate level. Higher knowledge scores were positively associated with more favorable attitudes and practices. However, the correlation coefficients indicated weak‐to‐moderate associations, and the relationships should be interpreted cautiously. Furthermore, regular physical exercise was significantly associated with higher knowledge and more positive attitudes knowledge and attitudes toward sarcopenia. These findings support the need for targeted education and for encouraging physical activity as part of routine care, which may be associated with better sarcopenia‐related awareness and self‐reported preventive practices.

This study corroborates observations made by healthcare professionals [23–25], further illustrating that knowledge gaps mainly involved diagnostic approaches and awareness of relevant comorbidities, often misconstruing it as an inevitable aspect of aging due to its subtle clinical manifestations. Knowledge shortfalls were most apparent in how sarcopenia is identified and what it implies clinically, suggesting that patient education should move beyond definitions toward actionable screening and risk information. Furthermore, it is crucial to elucidate the broader systemic ramifications of sarcopenia, including its significant connection to increased cardiovascular risks and its potential implications for insulin therapy. This focused content is essential for empowering patients to pursue early detection and proactive management. A scoping review similarly highlighted the limited awareness and knowledge of sarcopenia among healthcare professionals [26].

The findings suggest that unmarried individuals, as well as those with lower educational attainment and income levels, consistently exhibited inadequate KAP. This observation is consistent with previous research [27], which underscores the impact of socioeconomic conditions on health literacy. Beyond merely identifying these disparities, this study accentuates the urgent necessity for health education strategies that prioritize equity. Interventions targeting these vulnerable populations should actively address accessibility barriers and consider variations in information processing. Effective strategies may encompass community‐based outreach programs, the utilization of highly visual and easily comprehensible educational materials, and the dissemination of information through trusted local channels to bridge existing knowledge and access gaps. This is particularly significant for improving health outcomes among older patients, who frequently face considerable challenges in accessing and retaining information. Beyond these socioeconomic disparities in patient awareness and engagement, our data also point to a separate but equally important gap at the clinical level, namely the limited recognition and diagnosis of sarcopenia in routine diabetes care.

A significant finding from this study, representing a substantial new contribution, is the potential widespread underdiagnosis of sarcopenia within clinical practice. Despite the high risk of sarcopenia in patients with T2DM, the remarkably low rate of physician‐diagnosed cases in our cohort—only 2.53%—strongly suggests that sarcopenia may be largely overlooked in routine diabetes management rather than being truly rare. Initially perceived as a limitation for specific analyses, this finding actually highlights a critical gap: the apparent absence of routine screening and formal diagnosis of sarcopenia, even among high‐risk populations such as those with T2DM. Possible contributors include limited clinical awareness and the absence of routine screening pathways in diabetes care, which may lead sarcopenia to be overlooked until functional decline becomes apparent. These findings support integrating brief screening and counseling on muscle health into routine T2DM management, particularly for older adults.

Contrary to expectations, participants who engaged in exercise for more than 60 min per week demonstrated significantly higher knowledge scores regarding sarcopenia, yet exhibited lower attitude scores. A notable finding was the observation of a “knowledge‐attitude paradox,” in which participants with higher sarcopenia knowledge reported lower attitude scores in our measure. This pattern suggests that greater knowledge did not correspond to more positive attitudes in this subgroup and that a simple linear KAP assumption may not fully capture the underlying mechanisms. One possible explanation may be rooted in the concept of self‐efficacy: highly active individuals engaged in preventive behaviors might develop a strong sense of personal capability and control over their health. This increased self‐efficacy could result in a diminished perception of personal risk or urgency, consequently leading to lower scores on attitude scales that measure concern or perceived need for additional action.

This discrepancy may be partially attributable to the limitations inherent in the instruments used to measure attitudes. Attitude scales, particularly those employing Likert‐type items, may reflect changes in perceived risk rather than a true decline in positive sentiment. For instance, physically active individuals may agree with statements such as “I do not believe I am in a high‐risk group” because they are already engaging in preventive behaviors, which could lower their attitude scores based on the scoring mechanism. Such responses do not necessarily indicate negative attitudes, but rather a realistic appraisal of their current risk status and a belief that their proactive efforts are sufficient. This pattern may reflect lower perceived susceptibility among individuals already engaging in regular activity, and it may also be influenced by the scoring of attitude items related to perceived risk. Further qualitative work could clarify how physically active patients interpret sarcopenia risk and prevention needs.

Participants exhibited a positive attitude toward the prevention and management of sarcopenia, generally acknowledging the significance of a healthy diet and moderate exercise in preventing the condition [16]. Despite positive attitudes, a substantial intention–behavior gap was observed regarding preventive practices [28, 29]. This gap was further influenced by identified barriers such as financial costs, geographical proximity to facilities, and perceived lengthy treatment durations. As this is a patient‐reported KAP study, the following points are presented as implications for practice and policy rather than direct conclusions about clinical workflows or health system performance.

These findings imply that clinicians may consider actively screen for sarcopenia risk in patients with T2DM utilizing validated tools such as SARC‐F [30], with particular attention to older patients given their elevated risk. In addition to general advice, healthcare professionals should provide tailored prescriptions for exercise and nutrition, focusing on specific diagnostic methods and the systemic comorbidities of sarcopenia, including its association with cardiovascular disease. Equipping clinicians with standardized screening tools and brief counseling strategies is essential, as patients heavily rely on their guidance. The significant underdiagnosis of sarcopenia supports considering advocates for the integration of sarcopenia screening into routine T2DM management.

At a broader level, our findings suggest potential value in incorporating sarcopenia prevention education into community chronic disease management programs. Health education strategies prioritizing equity are vital for vulnerable populations—such as unmarried individuals and those with lower educational attainment and income levels—requiring culturally sensitive interventions delivered through accessible channels like community centers and digital platforms. It is crucial to address practical barriers by promoting affordable community exercise programs, expanding telehealth services, and improving insurance coverage for sarcopenia screenings [3, 18]. Public health campaigns should emphasize the multifaceted benefits of physical activity, including psychological factors like enhanced self‐efficacy and motivation, especially for the well‐being of the rapidly growing older adult population.

This study has several limitations. First, it was conducted at a single university‐affiliated hospital in China, the findings may predominantly reflect the characteristics of patients seeking care in a tertiary hospital, who might differ from those in community settings or other healthcare facilities concerning socioeconomic status, education, and disease complexity. This limitation in generalizability suggests that the findings may not fully reflect the broader population of individuals with T2DM, and they should be interpreted with caution, especially when applied to older adults in varied community settings. Future work should include multicenter and community‐based studies to confirm these results in more heterogeneous groups.

Second, the cross‐sectional design limits the ability to draw causal inferences between variables, allowing only the identification of associations. For instance, although the SEM specified hypothesized pathways among knowledge, attitude, practice, and exercise, the estimated paths should be interpreted as associations consistent with the proposed model, and we cannot ascertain the temporal sequence of these relationships.

It is plausible that individuals who already exercise regularly are more inclined to seek out and retain information about sarcopenia, rather than increased knowledge directly prompting greater participation in exercise. Moreover, the cross‐sectional nature of the study prevents evaluation of the long‐term impact of potential interventions on improving patients’ KAP levels related to sarcopenia. In addition, we did not collect detailed indicators of diabetes duration or glycemic severity, which are associated with sarcopenia risk and functional decline, and their omission may have introduced residual confounding in the observed relationships between KAP and related factors.

Third, data on practice behaviors were obtained through self‐reported questionnaires, which are inherently vulnerable to recall bias and social desirability bias. Participants may have exaggerated positive health behaviors or downplayed less desirable ones to align with perceived social expectations. Although the anonymous nature of the survey aimed to mitigate social desirability bias, it cannot be entirely eradicated. Nonetheless, self‐report surveys remain the most feasible and widely accepted method for assessing KAP in large populations, and the findings still provide valuable insights into patients’ perceptions and behaviors.

Finally, the low number of participants formally diagnosed with sarcopenia, previously discussed as indicative of underdiagnosis, also limited our ability to draw meaningful conclusions regarding KAP differences between diagnosed and undiagnosed individuals in our cohort. The KAP scores of the small group of diagnosed individuals may not be sufficiently representative for comparative analysis.

Future investigations should employ longitudinal designs to clarify the causal relationships between changes in KAP and the onset or progression of sarcopenia. Randomized controlled trials (RCTs) are essential for rigorously evaluating the effectiveness of specific educational interventions, particularly those targeting vulnerable populations and older patients. Additionally, qualitative research, such as in‐depth interviews, is vital for further examining the complex interactions between high knowledge, active engagement, and perceived attitudes, especially concerning self‐efficacy and risk perception among highly active individuals. Understanding these psychological factors will enhance the ability to develop effective behavioral strategies for sustained health promotion.

These findings provide important implications for designing sarcopenia interventions among patients with T2DM. The knowledge–attitude paradox observed in this study indicates that increasing knowledge alone is insufficient to enhance motivation or preventive behaviors. Guided by TPB and SCT, future interventions should incorporate tailored strategies that address self‐efficacy and perceived behavioral control. For individuals with low awareness and limited confidence, structured education combined with supervised exercise support may help strengthen attitudes and facilitate behavior change. Conversely, for those already physically active and exhibiting high perceived control, strategies such as reinforcing risk perception, personalized goal setting, and feedback on functional outcomes may be more effective in sustaining motivation. These results highlight that a uniform approach may not work for all patients; instead, differentiated and theory‐informed interventions are needed to translate KAP improvements into sustained practice.

Overall, our findings suggest a gap between sarcopenia risk and clinical recognition among adults with T2DM, and suggest that enhancing awareness by itself may be insufficient to convert positive attitudes into sustained preventive behaviors. Embedding brief, practical sarcopenia education and simple screening pathways into routine diabetes follow‐up, together with tailored exercise and nutrition guidance, may help close the intention–behavior gap in this high‐risk population. Future studies should test scalable, targeted interventions across diverse clinical and community settings and evaluate their impact on screening uptake and functional outcomes, while recognizing that the cross‐sectional design, reliance on self‐reported measures, and use of a single‐center sample may restrict causal inference and limit generalizability.

## 5. Conclusion

This study indicated that patients with type 2 diabetes possess limited knowledge, exhibit positive attitudes, and demonstrate moderate practices regarding sarcopenia. A significant concern arises from the underdiagnosis of sarcopenia in clinical settings, particularly among older patients, as this condition substantially impacts their quality of life and clinical outcomes. Consequently, there is an urgent necessity for targeted, equity‐focused educational interventions aimed at enhancing knowledge, addressing practical barriers, and translating positive attitudes into sustained healthy behaviors. Future qualitative research is imperative for investigating the psychological factors that influence health behaviors and for developing more effective, patient‐centered strategies for the prevention and management of sarcopenia.

**Nomenclature**
KAP: Knowledge, attitudes, and practices
T2DM: Type 2 diabetes mellitus
ANOVA: Analysis of variance.

## Author Contributions

Xiaodi Sun, Song Han, and Min Wang carried out the studies, participated in collecting data, and drafted the manuscript. Wen Liu and Zengqiang Liu performed the statistical analysis and participated in its design. Xiaodi Sun, Weidong Jiang and Aiqiong Qin participated in acquisition, analysis, or interpretation of data and draft the manuscript. All authors read and approved the final manuscript.

## Funding

No funding was received for this manuscript.

## Ethics Statement

This study was approved by the Ethic Committee of the Second Hospital of Shandong University (KYLL‐2023LW035). I confirm that all methods were performed in accordance with the relevant guidelines. All procedures were performed in accordance with the ethical standards laid down in the 1964 Declaration of Helsinki and its later amendments, and informed consent was obtained from all participants.

## Consent

Written informed consent was obtained from the patient for publication of this case report and any accompanying images.

## Conflicts of Interest

The authors declare that they have no competing interests.

## Supporting Information

Additional supporting information can be found online in the Supporting Information section.

## Supporting information


**Supporting Information** Table S1 Distribution of Knowledge Dimension.Table S2 Distribution of Attitudes Dimension. Table S3. Distribution of Practice Dimension. Table S4. Model fit indices of structural equation model.

## Data Availability

All data generated or analyzed during this study are included in this article and Supporting Information files.
