# Supplementary material for: Knowledge, Attitudes, and Practices About Sarcopenia in Adults With Type 2 Diabetes: A Cross‐Sectional Survey
Source: J Diabetes Res. 2026 Apr 23;2026:3057863. doi: 10.1155/jdr/3057863 (PMC13106980; doi:10.1155/jdr/3057863)
Supplement: Supplementary file 1 — Supporting Information Table S1 Distribution of Knowledge Dimension.Table S2 Distribution of Attitudes Dimension. Table S3. Distribution of Practice Dimension. Table S4. Model fit indices of structural equation model. [file JDR-2026-3057863-s001.docx]

**Supplementary Table 1 Distribution of Knowledge Dimension.**

| **Terms of Knowledge Dimension** | **Correctness Number (ratio, %)** |
| --- | --- |
| K1: Sarcopenia, also known as 'Muscle attenuation syndrome', is a condition of age-related loss of muscle mass. (Correct) | 388 (81.68) |
| K2: Sarcopenia only causes a loss of muscle strength and has no effect on physical function. (Incorrect) | 50 (10.53) |
| K3: The elderly population is a vulnerable group for sarcopenia. (Correct) | 398 (83.79) |
| K4: Anxiety and depression are a manifestation of sarcopenia. (Incorrect) | 22 (4.63) |
| K5: Screening for sarcopenia requires assessment of gait speed. (Correct) | 79 (16.63) |
| K6: Sarcopenia increases the risk of developing cardiovascular disease. (Correct) | 191 (40.21) |
| K7: Sarcopenia causes slow gait and reduced balance in patients with diabetes mellitus. (Correct) | 297 (62.53) |
| K8: Supplementing with high-quality protein can improve sarcopenia symptoms. (Correct) | 239 (50.32) |
| K9: Endurance training is the first-line exercise prescription for the treatment of sarcopenia. (Incorrect) | 12 (2.53) |
| K10: Patients with diabetes mellitus are more likely to develop sarcopenia than the general population. (Correct) | 409 (86.11) |
| K11: Patients with diabetes mellitus treated with insulin injections can induce sarcopenia. (Incorrect) | 28 (5.89) |
| K12: Patients with diabetes mellitus can be prevented from developing sarcopenia by eating more staple foods. (Incorrect) | 48 (10.11) |

**Supplementary Table 2 Distribution of Attitudes Dimension.**

| **Terms of Attitudes Dimension** | **Strongly agree** | **Agree** | **Neutral** | **Disagree** | **Strongly disagree** |
| --- | --- | --- | --- | --- | --- |
| A1: You think that ensuring muscle health is important for overall health. | 387 (84.68) | 60 (13.13) | 9 (1.97) | 1 (0.22) | 0 (0) |
| A2: You think that as patients with type 2 diabetes mellitus, muscle health is important to ensure independent living. | 387 (84.68) | 64 (14) | 6 (1.31) | 0 (0) | 0 (0) |
| A3: You think that proper physical activity is important to ensure muscle health. | 400 (87.53) | 45 (9.85) | 12 (2.63) | 0 (0) | 0 (0) |
| A4: You think that a balanced diet and nutritional intake is important to ensure muscle health. | 387 (84.68) | 62 (13.57) | 8 (1.75) | 0 (0) | 0 (0) |
| A5: You think that positive and well control of glucose can reduce the incidence of sarcopenia in diabetes. | 374 (81.84) | 70 (15.32) | 11 (2.41) | 1 (0.22) | 1 (0.22) |
| A6: You think you are in the group with a high prevalence of sarcopenia. | 277 (60.75) | 101 (22.15) | 23 (5.04) | 30 (6.58) | 25 (5.48) |
| A7: You think that it should perform regular lectures, training and screening groups at high risk of sarcopenia. | 375 (82.06) | 69 (15.1) | 13 (2.84) | 0 (0) | 0 (0) |
| A8: You think that a person with sarcopenia should seek help from a physician in time. | 381 (83.37) | 63 (13.79) | 10 (2.19) | 3 (0.66) | 0 (0) |
| A9: You are concerned that the treatment of sarcopenia may have adverse effects that make you fearful. | 159 (34.87) | 175 (38.38) | 57 (12.5) | 60 (13.16) | 5 (1.1) |
| A10: You think the sarcopenia treatment options provided by your doctor are trustworthy. | 371 (81.36) | 71 (15.57) | 12 (2.63) | 2 (0.44) | 0 (0) |
| A11: You are worried that having sarcopenia will reduce your quality of life. | 374 (81.84) | 70 (15.32) | 11 (2.41) | 2 (0.44) | 0 (0) |
| A12: You are worried that having sarcopenia will increase the burden on your family. | 369 (80.74) | 71 (15.54) | 13 (2.84) | 4 (0.88) | 0 (0) |

**Supplementary Table 3. Distribution of Practice Dimension.**

| **Terms of Practice Dimension** | **Always** | | **Often** | | | **Sometimes** | | **Rarely** | | **Never** | |
| --- | --- | --- | --- | --- | --- | --- | --- | --- | --- | --- | --- |
| P1: The frequency that you proactively understand and learn about sarcopenia. | 4 (0.84) | | 4 (0.84) | | | 19 (4) | | 263 (55.37) | | 185 (38.95) | |
| P2: The frequency that you proactively participate in lectures and trainings related to sarcopenia. | 2 (0.42) | | 5 (1.05) | | | 8 (1.68) | | 50 (10.53) | | 410 (86.32) | |
| P3: The frequency that you proactively participate in sarcopenia screening. | 2 (0.42) | | 4 (0.84) | | | 12 (2.53) | | 41 (8.63) | | 416 (87.58) | |
| P4: The frequency that you perform a sufficient amount of moderate-to-high intensity exercise per week. | 7 (1.48) | | 32 (6.75) | | | 122 (25.74) | | 255 (53.8) | | 58 (12.24) | |
| P5: The frequency that you keep a normal diet and balanced nutritional intake. | 291 (61.39) | | 105 (22.15) | | | 56 (11.81) | | 15 (3.16) | | 7 (1.48) | |
| P6: You can strictly follow your doctor's prescription for the treatment of type 2 diabetes and sarcopenia. | 345 (72.63) | | 77 (16.21) | | | 31 (6.53) | | 14 (2.95) | | 8 (1.68) | |
| P7: You will deliver proper diet, exercise and other sarcopenia prevention strategies to other patients with type 2 diabetes. | 335 (70.53) | | 49 (10.32) | | | 36 (7.58) | | 25 (5.26) | | 30 (6.32) | |
| P8: If you were unfortunate to develop sarcopenia, which forms of treatment would you prefer to start with? | 3 times a week of moderate to high intensity exercise training for 3 months | | High protein diet at breakfast, lunch and dinner | | | Both | | Neither | | Other | |
|  | 17 (3.58) | | 60 (12.63) | | | 352 (74.11) | | 42 (8.84) | | 4 (0.84) | |
| P9: If you needed to undergo sarcopenia treatment, what do you think would be a concern or a hindrance for you? (Multiple choice) | Too long duration of treatment | Too far from home and inconvenient to consult a doctor | | Too expensive treatment options to afford | The sarcopenia is not severe enough to be treated in hospital | | Terrified of going to the hospital for check-ups | Worried that the treatment of sarcopenia will affect the progression of type 2 diabetes | No concerns or hindrance | | Other |
|  | 209(44.00) | 313(65.89) | | 324(68.21) | 48(10.11) | | 32(6.74) | 150(31.58) | 32(6.74) | | 6(1.26) |
| P10: In terms of methods of popularising knowledge about sarcopenia, which forms of advocacy and education would be acceptable to you? (Multiple choice) | Scientific videos | | Health knowledge lectures | | | Brochures | | Science knowledge delivery via WeChat Public Accounts and other new media | | Other | |
|  | 243(51.16) | | 425(89.47) | | | 233(49.05) | | 201(42.32) | | 10(2.11) | |

**Supplementary table 4. Model fit indices of structural equation model.**

| **Fit** | **Ref.** | **Measured results** |
| --- | --- | --- |
| **CMIN/DF** | 1-3 excellent，3-5 good | 4.618 |
| **NFI** | >0.8 good | 0.961 |
| **IFI** | >0.8 good | 0.969 |
| **TLI** | >0.8 good | 0.882 |
| **CFI** | >0.8 good | 0.969 |
